# Effects of Vitamin D_3_ and Meso-Zeaxanthin on Human Retinal Pigmented Epithelial Cells in Three Integrated *in vitro* Paradigms of Age-Related Macular Degeneration

**DOI:** 10.3389/fphar.2021.778165

**Published:** 2021-11-05

**Authors:** Francesca Lazzara, Federica Conti, Chiara Bianca Maria Platania, Chiara M. Eandi, Filippo Drago, Claudio Bucolo

**Affiliations:** ^1^ Department of Biomedical and Biotechnological Sciences, School of Medicine, University of Catania, Catania, Italy; ^2^ Department of Ophthalmology, Fondation Asile des Aveugles, Jules Gonin Eye Hospital, University of Lausanne, Lausanne, Switzerland; ^3^ Department of Surgical Sciences, University of Torino, Torino, Italy; ^4^ Center for Research in Ocular Pharmacology–CERFO, University of Catania, Catania, Italy

**Keywords:** 1,25(OH)_2_D_3_, meso-zeaxanthin, amyloid beta, inflammation, oxidative stress, cytokines

## Abstract

Age-related macular degeneration (AMD) is a degenerative retinal disease and one of major causes of irreversible vision loss. AMD has been linked to several pathological factors, such as oxidative stress and inflammation. Moreover, Aβ (1–42) oligomers have been found in drusen, the extracellular deposits that accumulate beneath the retinal pigmented epithelium in AMD patients. Hereby, we investigated the hypothesis that treatment with 1,25(OH) _2_D_3_ (vitamin D_3_) and meso-zeaxathin, physiologically present in the eye, would counteract the toxic effects of three different insults on immortalized human retinal pigmented epithelial cells (ARPE-19). Specifically, ARPE-19 cells have been challenged with Aβ (1–42) oligomers, H_2_O_2_, LPS, and TNF-α, respectively. In the present study, we demonstrated that the combination of 1,25(OH)_2_D_3_ and meso-zeaxanthin significantly counteracted the cell damage induced by the three insults, at least in these *in vitro* integrated paradigms of AMD. These results suggest that combination of 1,25(OH)_2_D_3_ and meso-zeaxathin could be a useful approach to contrast pathological features of AMD, such as retinal inflammation and oxidative stress.

## Introduction

Age-related macular degeneration (AMD) is a progressive neurodegenerative and multifactorial disease that if not treated or managed can impair irreversibly the visual function (Cascella et al., 2014; Pennington and DeAngelis, 2016) in the elderly population (usually older than 60 years) (Nowak, 2006). AMD affects the macula, that is, the central portion of the retina, which is highly sensitive to visual stimuli due to the high density of retinal photoreceptors. In the macula of AMD patients, between the retinal pigment epithelium (RPE) and Bruch’s membrane, lesions named *drusen* have been found. These lesions are characterized by accumulation of extracellular material, lipid, and protein aggregates. Moreover, the number and size of drusen, along with the presence of choroidal neovascularization, have been found to correlate with the stage of AMD (early, intermediate, or advanced) (Zajaç-Pytrus et al., 2015). Generally, AMD is classified into atrophic (dry or non-exudative form) and neovascular or exudative forms (wet form). Wet AMD is characterized by overexpression of the vascular endothelial growth factor (VEGF-A), which leads to the breakdown of the blood–retinal barrier and choroidal neovascularization (Kauppinen et al., 2016). Retinal degeneration in wet AMD is tightly linked to choroidal neovascularization (CNV) and growth of leaky blood vessels under the macula, due to overproduction of pro-angiogenic factors (VEGF family) and inflammatory cytokines. Dry AMD can progress to the severe stage, wet AMD, which if not managed can lead to macular edema, retinal detachment, and then to irreversible blindness. Actually, only patients with the wet form (neovascular AMD) can be benefitted from pharmacological therapy, specifically the intravitreal administration of anti-vascular endothelial growth factors (anti-VEGF) (Holekamp, 2019), although anti-VEGF agents, used in clinical practice, such as ranibizumab, bevacizumab, and aflibercept, are considerably different in terms of molecular interactions when they bind with VEGF (Giurdanella et al., 2015; Platania et al., 2015). Currently, one of the main unmet medical needs in AMD management is the lack of effective pharmacological treatment for the dry AMD, which represents the 90% of AMD cases (Buschini et al., 2015). Moreover, the pathophysiology of the AMD is only partially understood, considering that it is the result of the interaction between environmental, metabolic, and genetic factors. Main hallmarks of AMD are represented by tissue dysfunctions (RPE, Bruch’s membrane, and choriocapillaris), associated to chronic oxidative stress, autophagy decline, inflammation (Levy et al., 2015; Eandi et al., 2016; Guillonneau et al., 2017), and angiogenesis (Kauppinen et al., 2016; Layana et al., 2017). Several studies highlighted that inflammation is one of the main driving factors of AMD pathogenesis. In fact, drusen deposits contain numerous inflammation-related factors, along with lipids, amyloid-β (Aβ) aggregates, and oxidation by-products (Bucolo et al., 1999; Wang et al., 2009; Krohne et al., 2010). Furthermore, it has been demonstrated that the formation of drusen is induced by chronic low-level inflammation and complement activation, as a result of the activation of inflammatory pathways, such as NFκB (Hageman et al., 2001; D.H. et al., 2002; Johnson et al., 2011). Moreover, the activation of the inflammasome, by amyloid-β, was reported to contribute to RPE dysfunction during AMD (Anderson et al., 2013; Liu et al., 2013). Macrophages, attracted by drusen to the sub-RPE space, release tumor necrosis factor α (TNF-α) that binds tumor necrosis factor receptor 1 (TNFR1), and then stimulate RPE cells’ inflammatory response. AMD is also known as the “dementia of the eye,” due to the age-dependent accumulation of amyloid beta oligomers in drusen deposits. Several studies demonstrated that Aβ-related damage is common to both the retina and brain, as well as the disruption of the tight junctions in the blood–brain barrier (BRB) and the blood–retinal barrier (BRB) (Parks et al., 2004; Bruban et al., 2009; Biron et al., 2011). Together with inflammation and Aβ-related damage, reactive oxygen species (ROS) have a central role in AMD (Kohen and Nyska, 2002). The altered cellular homeostasis in RPE cells, related to ROS overproduction, can be induced by several factors, such as, aging process, light exposure, diet, and cigarette smoking.

Indeed, because of the multifactorial pathophysiology of both dry and wet AMD, we designed an integrated *in vitro* model of AMD, stimulating RPE cells with three different challenges: Aβ oligomers, hydrogen peroxide (H_2_O_2_), and inflammatory stimuli (LPS and TNF-α), and testing the effects of *in vitro* treatment with anti-inflammatory, anti-angiogenic, and antioxidant molecules: 1,25(OH)_2_D_3_ (vitamin D_3_), meso-zeaxanthin (MZ), and their combination. Specifically, vitamin D_3_ is a secosteroid able to modulate cell differentiation, homeostasis, and apoptosis through direct and indirect mechanisms of action. The first one is activated by the binding of the active form of vitamin D_3_ to its receptor (VDR), a transcriptional factor. VDR is expressed in most human cells, supporting the hypothesis that vitamin D_3_ has a pleiotropic effect. Moreover, anti-inflammatory and anti-angiogenic effects of vitamin D_3_ have been widely elucidated both in *in vitro* and *in vivo* studies (Majewski et al., 1996; Albert et al., 2007; Maj et al., 2018; Almeida Moreira Leal et al., 2020). Interestingly, the vitamin D_3_ receptor is expressed in the RPE layer, which along with enzymes is able to convert the inactive form into the active form. The rationale of this *in vitro* study came from previous reports that have shown a tight link between vitamin D_3_ serum levels and AMD progression. In fact, it has been found that a low vitamin D_3_ level in serum can be a risk factor for the progression of AMD (Parekh et al., 2007; Millen et al., 2011; Annweiler et al., 2016; Merle et al., 2017; Kan et al., 2020). These findings could be linked to the activation of macrophages phagocytosis of Aβ deposits, along with anti-inflammatory and antioxidant action exerted by vitamin D_3_ (Lee et al., 2012).

Meso-zeaxanthin [(3R, 30S)-b, b-carotene-3, 30-diol, MZ] is one of the three xanthophyll carotenoids localized in the *macula lutea*. Carotenoids are lipid-soluble yellow–orange–red pigments with antioxidant and immunomodulatory activity; reduction in carotenoid levels has been linked to increased risk of cardiovascular disease, diabetes, and cancer (Sesso et al., 2004; Hozawa et al., 2006; Eliassen et al., 2015). In particular, MZ is one of the powerful antioxidant carotenoids found in the RPE cell layer. Basically, the source of meso-zeaxanthin in the eye is represented by the endogenous conversion of lutein in the retinal pigment epithelium (Shyam et al., 2017; Green-Gomez et al., 2020). A specific carotenoid-binding protein (Z-binding protein) regulates the retinal uptake from blood of lutein, which can be converted into meso-zeaxanthin (Thurnham et al., 2008; Nolan et al., 2013).

Given these premises on vitamin D_3_ and meso-zeaxanthin activities, we tested the efficacy of these two compounds and their combination in three different *in vitro* models of AMD. We found that their combination significantly counteracted the damage induced by Aβ-amyloid oligomers, H_2_O_2_, and inflammatory stimuli in immortalized human RPE (ARPE-19) cells. Moreover, a bioinformatic analysis evidenced that the combination of these compounds effectively covers the pathways associated with the three stimuli, resembling the AMD multifactorial pathology.

## Methods

Human retinal pigment epithelial cells (ARPE-19) were purchased from ATCC® (Manassas, Virginia, USA). Cells were cultured at 37 °C (humidified atmosphere with 5% CO2) in ATCC-formulated DMEM:F12 medium (ATCC number 30–2006) with 100 U/ml penicillin, 100 μg/ml streptomycin, and 10% fetal bovine serum (FBS). After reaching confluence (70%), ARPE-19 cells were pretreated for 24 h with 50 nM of 1,25(OH)_2_D_3_ (Sigma Aldrich, D1530-1mg, St. Louis, MO), 0.1 µM of meso-zeaxanthin (MZ) (Sigma Aldrich, USP reference standard #1733119, St. Louis, MO), and the combination (combo) of 1,25(OH)_2_D_3_ (50 nM) and meso-zeaxanthin (MZ, 0.1 µM). Both pretreatment and treatment were performed in medium supplemented with 5% FBS to starve cells. After pretreatment, ARPE-19 cells were challenged with four different stimuli: amyloid-β oligomers (1 and 2.5 µM; amyloid β-protein 1–42 HFIP-treated, Bachem H-7442.0100) (Calafiore et al., 2012; Caruso et al., 2021), hydrogen peroxide (400 µM H_2_O_2_), LPS (150 ng/ml and 10 μg/ml, Enzo ALX-581–010-L001, Farmingdale, NY), and tumor necrosis-alpha (TNF-α) (10 ng/ml, Thermo Fisher Scientific, Carlsbad, CA), in order to simulate retinal degeneration, retinal oxidative stress, and early and late inflammation, respectively. 1,25(OH)_2_D_3_, MZ, and the combo were also added to the medium containing negative stimuli.

### Cell Viability

The 3-[4,5-dimethylthiazol-2-yl]-2,5-diphenyl tetrasodium bromide (MTT; Chemicon, Temecula, CA) was used to assess cell viability after Aβ (1–42) and H_2_O_2_ challenge. Optimal cell density was obtained by seeding 3 × 10^4^ cells/well in 96-well plates (Costar, Corning, NY, United States). After pretreatment, ARPE-19 cells were subjected to co-treatment in a fresh medium for 48 h with Aβ (1–42) (1 µM) and for 6 and 24 h with H_2_O_2_ (400 µM). At the end of the treatment, ARPE-19 cells were incubated at 37°C with MTT (0.5 mg/ml) for 3 h; then DMSO was added, and absorbance was measured at 570 nm in a plate reader (VariosKan, Thermo Fisher Scientific, Waltham, MA, United States). Graphs were built converting absorbance (abs) to viability (=% of control) using the following equation (absx ÷ absctrl−)  × 100, where absx is absorbance in the x well, and absctrl− is the average absorbance of negative control cells (untreated cells).

### Lactate Dehydrogenase Cell Release

Lactate dehydrogenase (LDH) cell release was measured using the Cytotoxicity Detection KitPLUS (LDH) (ROCHE, Mannheim, Germany). ARPE-19 cells were seeded at 2 × 10^4^ cells/well in 96-well plates (Costar, Corning, NY, United States). After pretreatment, cells were co-treated for 48 h with Aβ (1–42) (1 µM) and for 6 and 24 h in the oxidative stress model (H_2_O_2_ 400 µM). In control groups, only fresh medium was added. After these time points, according to manufacturer’s protocol, lysis solution was added to positive control wells (non-treated cells) for 15 min. After transferring 100 µl of medium in a new multi-well plate, 100 µl of working solution was added. After 10–15 min at room temperature, at last, 50 µl of stop solution was added. The absorbance values were measured at 490 nm using a plate reader (VarioSkan, Thermo Fisher Scientific, Waltham, MA, United States). LDH release is reported as LDH (% control) (absx  ÷  absctrl+)  ×  100. In the equation, abs_x_ is absorbance in the x well and abs_ctrl+_ is the average absorbance of positive control cells (untreated lysed cells). Absorbance values were corrected by subtracting medium absorbance.

### Reactive Oxygen Species Production

ROS were measured by a 2′,7′-dichlorofluoresceindiacetate (DCFDA)–Cellular Reactive Oxygen Species Detection Assay Kit (Abcam, Cambridge, United Kingdom). DCFDA, a cell permeable fluorogenic dye, is deacetylated by cellular esterases to a non-fluorescent compound and later oxidized by ROS to highly fluorescent 2′,7′-dichlorofluorescein (DCF); fluorescence intensity is proportional to cell ROS concentration. Optimal cell density was obtained by seeding 20 × 10^3^ cells/well in 96-well plates (Costar, Corning, NY, United States). After reaching confluence (70%), ARPE-19 cells were pretreated with 1,25(OH)_2_D_3_, mesozeaxanthin, and the combo for 24 h. Subsequently, cells were submitted to co-treatment for 48 h in amyloid-β challenge (1 µM). After treatment, media were aspirated and cells were washed by adding 100 µl/well of 1X buffer, according to manufacturer’s protocol; after washing, ARPE-19 cells were stained by adding 100 µl/well of the diluted DCDFA solution (25 µM). Cells were also incubated with this solution for 45 min at 37°C in the dark. After removing DCDFA solution, 100 µl/well of 1X buffer was added, and ROS concentration was measured immediately by detection of DCF fluorescence (λ_ex_ = 495 nm, λ_em_ = 529 nm) with a Varioskan™ Flash Multimode Reader. According to manufacturer’s protocol, for treatment lower than or equal to 6 h, it is possible to treat cells after adding DCDFA solution. Thus, after 24 h of pretreatment with drug formulations, ARPE-19 cells were washed and stained with DCDFA for 45 min. After removing DCDFA solution and washing again, ARPE-19 cells underwent co-treatment for 6 h in H_2_O_2_ challenge (400 µM). At the end of time point, ROS concentration was measured immediately without washing. Results were reported as percentage of control after background subtraction; to determine total ROS formation, the fluorescence was normalized to the fluorescent intensity of control cells (untreated cells).

### Extraction of Total Ribonucleic Acid and cDNA Synthesis

Extraction of total RNA, from ARPE-19 cells, was performed with a TRIzol Reagent (Invitrogen, Life Technologies, Carlsbad, CA, United States). The A_260_/A_280_ ratio of optical density of RNA samples (measured with Multimode Reader Flash di Varioskan™) was 1.95–2.01; this RNA purity was confirmed with the electrophoresis in non-denaturing 1% agarose gel (in TAE). cDNA was synthesized from 2 µg RNA with a reverse transcription kit (SuperScript™ II Reverse transcriptase, Invitrogen, Thermo Fisher Scientific, Carlsbad, CA, United States).

### Real-Time Reverse Transcriptase–Polymerase Chain Reaction

Real-time PCR was carried out with the Rotor-Gene Q (Qiagen). The amplification reaction mix included the Master Mix Qiagen (10 µl) (Qiagen QuantiNova SYBR Green Real-Time PCR Kit) and cDNA (1 µL, 100 ng). Forty-five amplification cycles were carried out for each sample. Results were analyzed with the 2^−ΔΔ^Ct method. Quantitative PCR experiments followed the MIQE guidelines. Gene expression levels were normalized with levels of two housekeeping genes (18S and GAPDH). Primers were purchased from Eurofins Genomics (Milan, Italy) and Qiagen (Milan, Italy). Forward and reverse primer sequences (for human genes) and the catalog number are herein listed: human IL-1β (forward: 5′-AGC​TAC​GAA​TCT​CCG​ACC​AC-3'; reverse: 5′-CGT​TAT​CCC​ATG​TGT​CGA​AGA​A-3′), human IL-6 (Catalog Number QT00083720), human TNF-α (forward 5′-AGC​CCA​TGT​TGT​AGC​AAA​CC-3'; reverse 5′-TGA​GGT​ACA​GGC​CCT​CTG​AT-3′), human MMP-9 (forward 5′-CTT​TGA​GTC​CGG​TGG​ACG​AT-3'; reverse 5′-TCG​CCA​GTA​CTT​CCC​ATC​CT-3′), human VEGF-A (forward 5′-AGG​GCA​GAA​TCA​TCA​CGA​AG-3'; reverse 5′-ATC​CGC​ATA​ATC​TGC​ATG​GT-3′), human 18S (forward 5′-AGT​CCC​TGC​CCT​TTG-3'; reverse 5′-GAT​CCG​AGG​GCC​TCA​CTA​AAC-3′), and human GAPDH (forward 5′-CTG​CAC​CAC​CAA​CTG​CTT​AG-3'; reverse 5′-AGG​TCC​ACC​ACT​GAC​ACG​TT-3′).

### Western Blot

ARPE-19 cells were cultured in 60-mm petri dishes at a density of 1.3 × 10^6^. After 24 h of pretreatment with drugs and co-treatment with different stimuli (400 µM of H_2_O_2_ for 4 h, 10 μg/ml of LPS for 2 h, amyloid-β oligomers 2.5 µM for 48 h, and TNF-α 10 ng/ml for 2 h), cytoplasmic and nuclear proteins were extracted by using the CER/NER kit (NE-PER, Invitrogen, Life Technologies, Carlsbad, USA), according to the manufacturer’s protocol. The protein content was determined by the BCA Assay Kit (Pierce™ BCA Protein Assay Kit, Invitrogen, Life Technologies, Carlsbad, United States). Extracted proteins (20 μg) were loaded on a NuPAGE ™ 10% Bis-Tris mini protein gel (Invitrogen, Life Technologies, Carlsbad, CA, United States). After electrophoresis, proteins were transferred to a nitrocellulose membrane (Invitrogen, Life Technologies, Carlsbad, CA, United States). Membranes were blocked with milk, 5% Tris-buffered saline, and 0.2% Tween 20 (TBST) for 1 h at room temperature. Membranes were incubated overnight (4°C) with appropriate primary phospho-NFκB p65 (Ser536; mouse mAb #3036 Cell Signaling Technology, MA, United States, 1:500 dilution), anti-GAPDH (Rabbit mAb #2118 Cell Signaling Technology, MA, United States; 1:1,000 dilution), and anti-lamin B (Mouse monoclonal IgG_2b_, sc-365214 Santa Cruz Biotechnology; 1:1,000 dilution) antibodies. After overnight incubation, the membranes were then incubated with secondary chemiluminscent antibodies (ECL anti-mouse, NA931 and ECL anti-rabbit, NA934, 1:2000 dilution) for 1 h at room temperature. After secondary antibody, the membranes were incubated with ECL (SuperSignal™ West Pico PLUS Chemiluminescent Substrate, Thermo Fisher Scientific, Carlsbad, CA, United States) and were detected through I-Bright™ 1500 (Invitrogen, Life Technologies, Carlsbad, CA, United States) by using chemiluminescence. Densitometry analyses of blots were performed at non-saturating exposures and analyzed using ImageJ software (NIH, Bethesda, MD). Values were normalized to GAPDH and lamin B, which were used as housekeeping control for cytoplasmic and nuclear fraction, respectively.

### Bioinformatics

The STITCH compound app of Cytoscape v. 3.7.0 was used to build an integrated network resembling all the experimental results obtained with our integrated *in vitro* model. Inputs were (i.e., query terms) β amyloid, LPS, TNF-α, H_2_O_2_, meso-zeaxanthin, vitamin D_3_, IL-6, Il-1β, VEGF-A, and MMP-9. The number of interactors was limited to 15, and the default confidence score was set to 0.40. Enrichment of information was included in the analysis. A centrality metrics analysis was carried out treating the network as an indirect graph (Platania et al., 2015, 2018). Functional clusters were identified with Cytoscape using specific terms: β amyloid, H_2_O_2_, LPS, TNF-α, vitamin D_3_, and meso-zeaxanthin.

### Statistical Analysis

Statistical analysis was performed with GraphPad Prism 7 (GraphPad software, La Jolla, California). All experiments were repeated five times (*n* = 5), and the data are reported as mean ± SD. One-way analysis of variance (ANOVA) was carried out, and Tukey’s post hoc test was used for multiple comparisons. Differences between groups were considered statistically significant for *p*-values < 0.05.

## Results

### Aβ-Oligomers Damage

**FIGURE 1:**
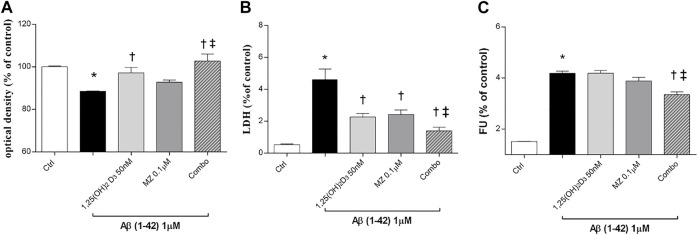
1,25(OH)_2_D_3_, meso-zeaxanthin (MZ), and their combination show protective effect in ARPE-19 cells treated with Aβ (1–42). Cells were pretreated for 24 h with tested compounds and for 48 h with Aβ insult. At the end of treatment were carried out MTT **(A)**, LDH **(B)**, and the ROS assay **(C)**. Values are reported as mean ± SD (*n* = 5). Data were analyzed by one-way ANOVA and Tukey’s post hoc test for multiple comparisons. *p < 0.05 vs. control; †p < 0.05 vs. Aβ, ‡p < 0.05 vs. 50 nM 1,25(OH)_2_D_3_ or 0.1 µM MZ.

In this study, we tested the protective effect of 1,25(OH)_2_D_3_, meso-zeaxanthin (MZ), and their combination against Aβ (1–42) oligomer-induced cytotoxicity, through measurement of ARPE-19 cell viability, after challenge with Aβ (Figure 1). Preliminary studies were carried out with the MTT assay to evaluate Aβ-oligomer toxicity on ARPE-19 cells, and we found that 1 μM Aβ-oligomers for 48 h induced roughly 17% cell death. Indeed, 1 μM Aβ-oligomers concentration was used also for LDH and ROS assays. In preliminary studies, ARPE-19 cells were pretreated with different concentrations of 1,25(OH)_2_D_3_, MZ, and their combination for 24 h. Therefore, cells were incubated with 1 μM Aβ for 48 h, the most effective compound concentrations were 50 nM and 0.1 μM for 1,25(OH)_2_D_3_ and MZ, respectively; indeed we used these concentrations also in the combination of the two compounds [combo: 1,25(OH)_2_D_3_ 50 nM + MZ 0.1 μM]. 1,25(OH)_2_D_3_ and the combo pretreament significantly (*p* < 0.05) counteracted cell toxicity induced by challenge with Aβ (MTT assay, Figure 1A). Moreover, LDH release was significantly increased (*p* < 0.05) after treatment with Aβ; the tested compounds 1,25(OH)_2_D_3_ and MZ, and their combination (combo), induced a significant (*p* < 0.05) reduction of cell damage after 48 h (Figure 1B). Finally, we analyzed the antioxidant activity of tested compounds. After 48 h of exposure, Aβ-oligomer insult significantly increased (*p* < 0.05) ROS release in ARPE-19 cells. Only the combination of 1,25(OH)_2_D_3_ and MZ was able to significantly reduce the amount of ROS after 48 h (*p* < 0.05) (Figure 1C), compared to Aβ-positive control cells.

**FIGURE 2:**
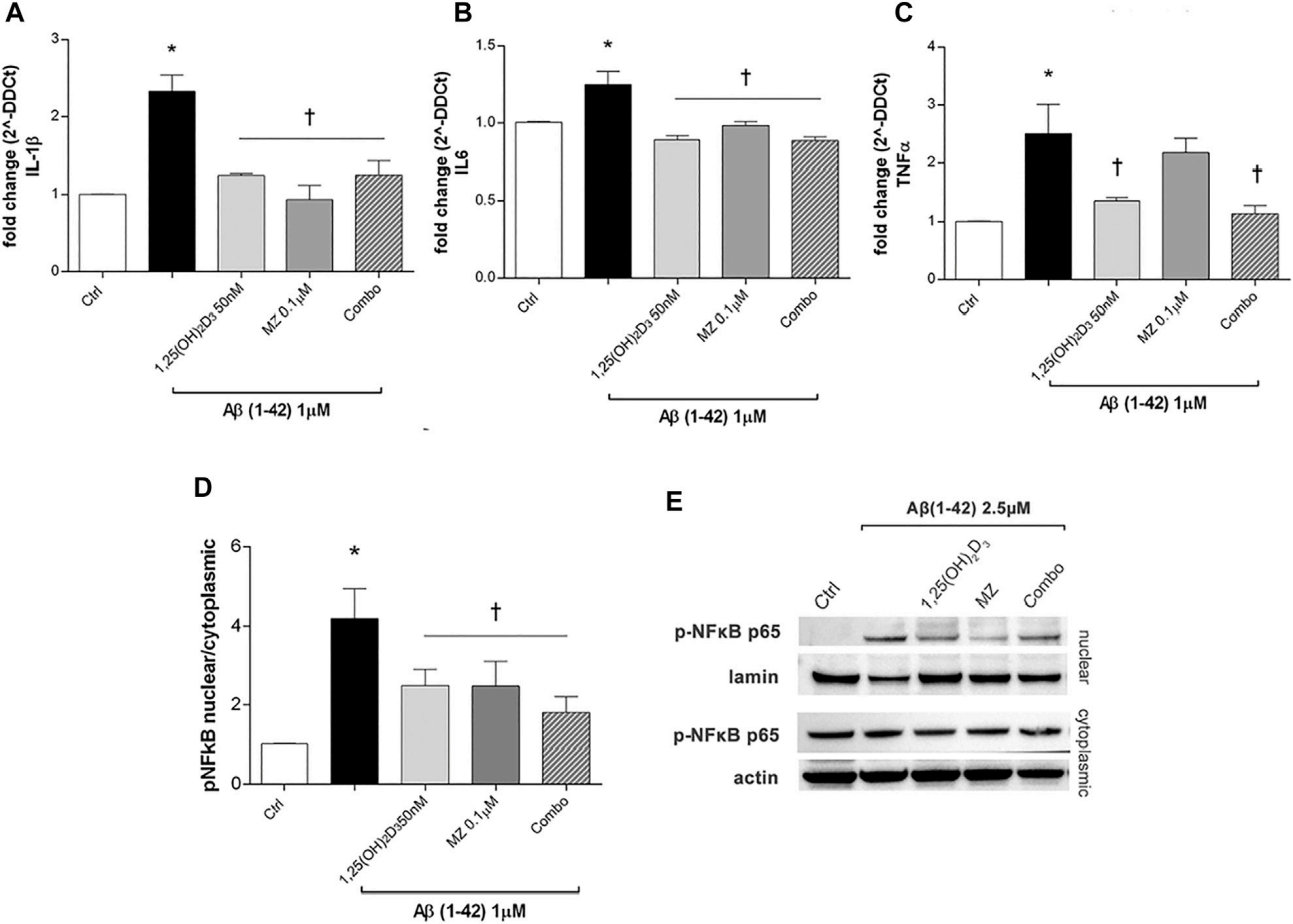
Treatment of ARPE-19 cells with 1,25(OH)_2_D_3_, meso-zeaxanthin (MZ), and their combination (combo) counteract inflammation after Aβ (1–42) exposure. The treatment with 1,25(OH)_2_D_3_, MZ, and their combo reduced IL-1β **(A)**, IL-6 **(B)**, and TNF-α **(C)** mRNA expression. The mRNA levels were evaluated by qPCR. **(D)** Western blot analysis. Densitometry analysis of each band (ratio of nuclear *p*-NFκB p-65/lamin B and cytoplasmic *p*-NFκB p-65/actin) was carried out with the ImageJ program. **(E)** Representative blots of nuclear and cytoplasmic extracted proteins from control and treated cells. Each bar represents the mean value ±SD (*n* = 5; each run in triplicate). One-way ANOVA and Tukey’s post hoc test for multiple comparisons were carried out. *p < 0.05 vs. control; †*p* < 0.05 vs. Aβ.

After 24h, Aβ oligomers exposure (1 µM) significantly (*p* < 0.05) increased mRNA expression of IL-1β, IL-6, and TNF-α (Figures 2A–C) in ARPE-19 cells. The treatment with 1,25 (OH)_2_D_3_, meso-zeaxanthin (MZ), and their combination significantly decreased IL-1β (Figure 2A) and IL-6 (Figure 2B), while only 1,25(OH)_2_D_3_ and the combo significantly reduced TNF-α mRNA expression (Figure 2C). Furthermore, Aβ treatment significantly (*p* < 0.05) increased nuclear translocation of p-NFκB p65 after 48 h of insult (Figure 2D). On the other hand, pretreatment for 24 h with 1,25(OH)_2_D_3_, MZ, and combo significantly (*p* < 0.05) reduced the translocation *p*-NFκB p65, confirming the anti-inflammatory effect of these two compounds and their combination, in retinal pigmented epithelial cells, challenged with Aβ oligomers (Figures 2D,E).

### Oxidative Stress

**FIGURE 3:**
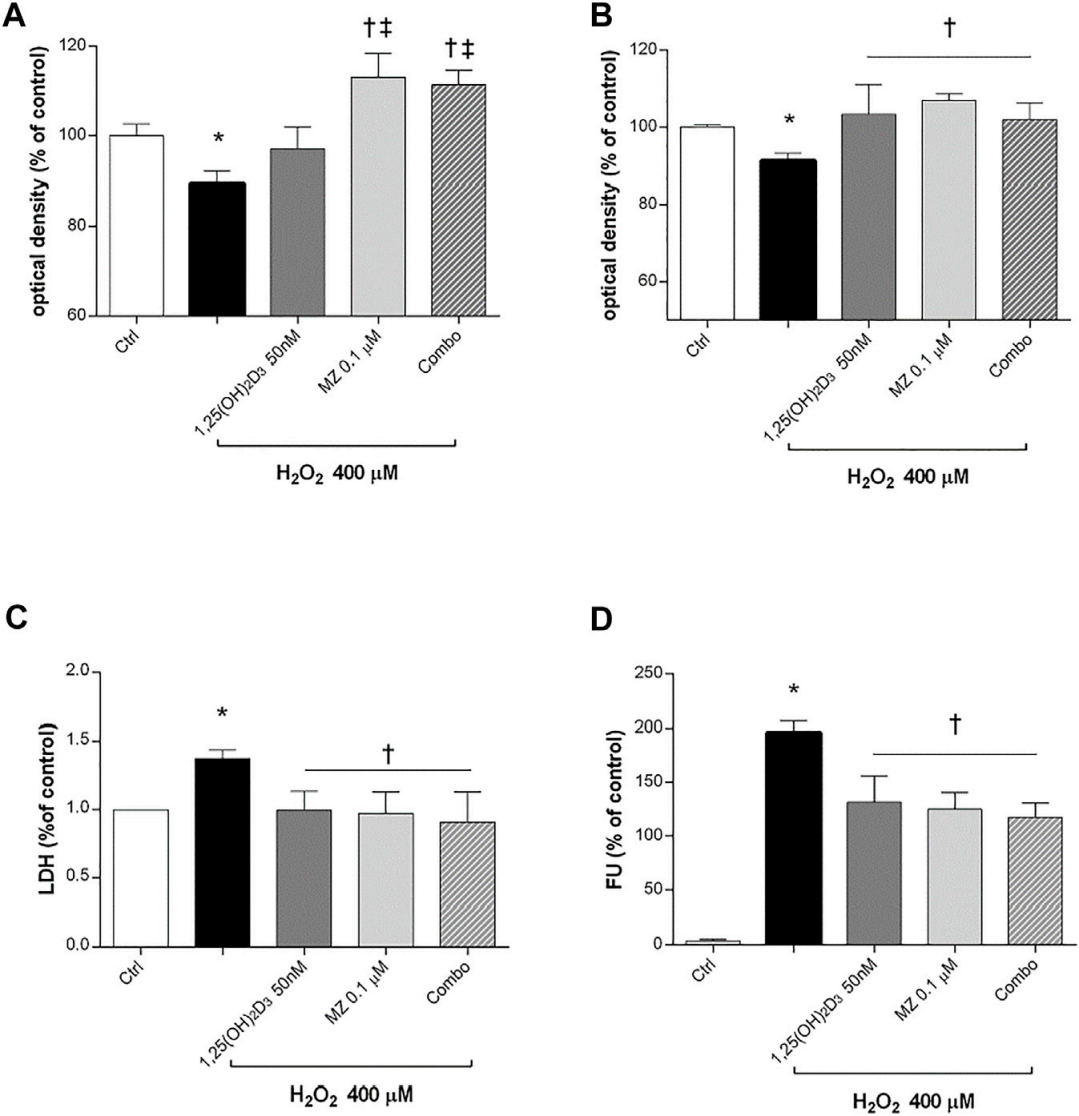
1,25(OH)_2_D_3_, meso-zeaxanthin (MZ), and their combination protect ARPE-19 cells from oxidative damage. ARPE-19 cells were pretreated for 24 h with 1,25(OH)_2_D_3_ (50 nM), MZ (0.1 µM), and their combo (1,25(OH)_2_D_3_ 50 nM, MZ 0.1 µM), and then treated with H_2_O_2_ (400 µM) for the MTT assay at 6 **(A)** and 24 h **(B). (C)** LDH release of ARPE-19 cells treated for 24 h with H_2_O_2_ (400 µM). **(D)** Pretreatment with 1,25(OH)_2_D_3_ (50 nM), MZ (0.1 µM), and their combination decreased ROS (fluorescent units, FU) production in ARPE-19 cells, challenged for 6 h with 400 µM H_2_O_2_. The results are expressed as mean ± SD (n = 5; each run in triplicate). Data were analyzed by one-way ANOVA and Tukey’s post hoc test for multiple comparisons. *p < 0.05 vs*.* ctrl; †*p* < 0.05 vs. H_2_O_2_; ‡p < 0.05 vs. 1,25(OH)_2_D_3_.

Preliminary studies on ARPE-19 cells were carried out to assess the best H_2_O_2_ concentration and time of exposure to oxidative stress able to elicit roughly 15% cell death. Therefore, human retinal pigmented epithelial cells were pretreated for 24 h with 1,25(OH)_2_D_3_ (50 nM), meso-zeaxanthin (MZ) (0.1 µM), and combo (1,25(OH)_2_D_3_ 50 nM, MZ 0.1 µM), and then cells were incubated in 400 µM H_2_O_2_ for 6 h (Figure 3A) and 24 h (Figure 3B). After 6 h of challenge, 1,25(OH)_2_D_3_ was not able to counteract H_2_O_2_-induced cell damage, instead after 24 h both compounds and their combination significantly restored cell viability. Moreover, H_2_O_2_ significantly (*p* < 0.05) increased LDH levels in ARPE-19 cells, and the pretreatment with tested compounds induced a significant reduction of cell damage (Figure 3C). Furthermore, we evaluated the effect of the tested compounds and their combination in terms of ROS production on ARPE-19 cells after H_2_O_2_ exposure. After 6h, H_2_O_2_ significantly increased (*p* < 0.05) ROS in ARPE-19 cells, compared to control cells (Figure 3D). Pretreatment with 1,25(OH)_2_D_3_, MZ, and their combination significantly (*p* < 0.05) counteracted oxidative stress in retinal cells, reducing ROS release.

**FIGURE 4:**
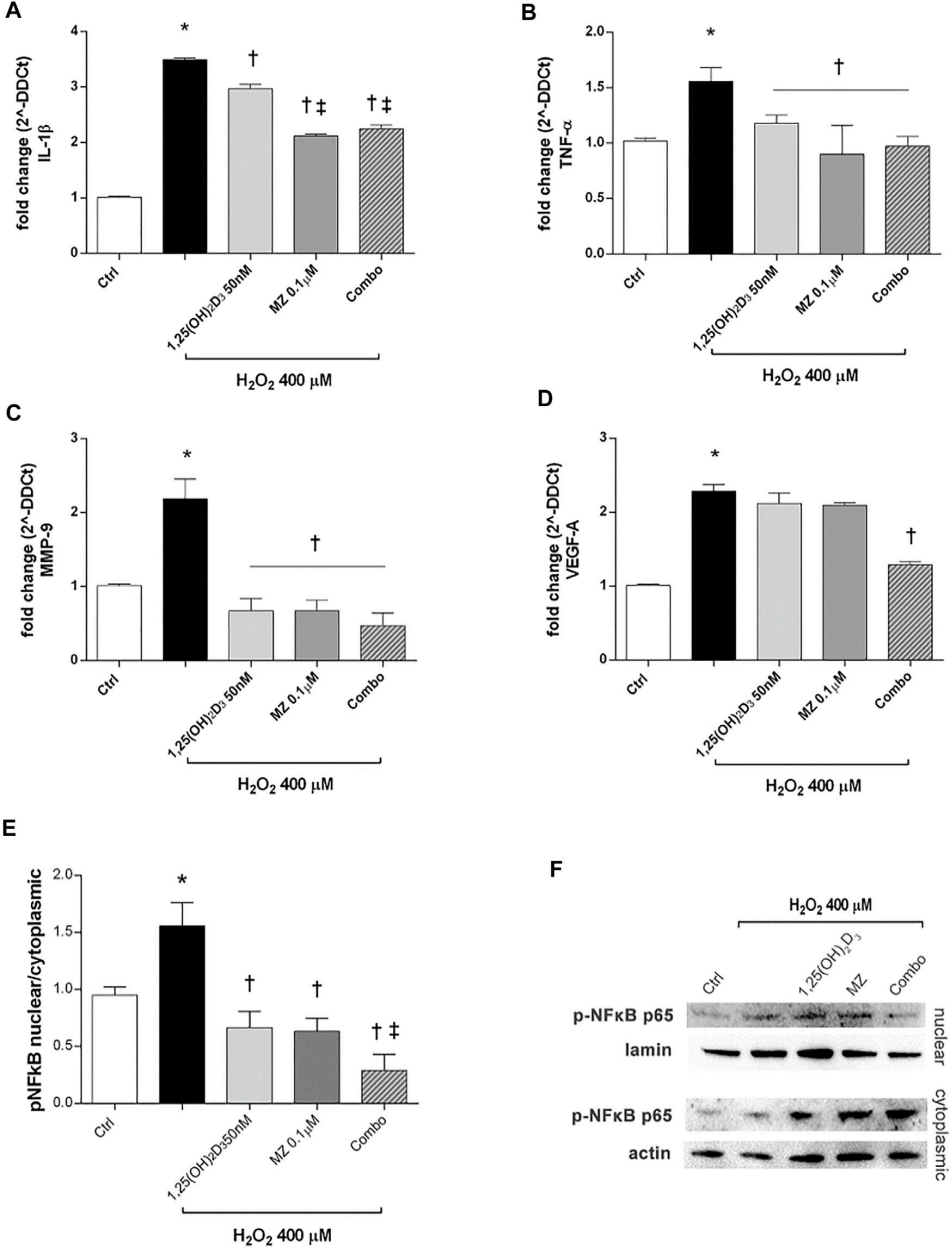
1,25(OH)_2_D_3_, MZ, and their combination attenuate H_2_O_2_-induced inflammation. 1,25(OH)_2_D_3_, MZ, and the combination reduced IL-1β **(A)**, TNF-α **(B)**, and MMP-9 **(C)** mRNA expression. The combo decreased VEGF-A mRNA expression induced after 6 h of H_2_O_2_ treatment **(D)**. ARPE-19 cells were pretreated for 24 h with 1,25(OH)_2_D_3_ (50 nM), MZ (0.1 µM), and their combo (1,25(OH)_2_D_3_ 50 nM + meso-zeaxanthin 0.1 µM), and then challenged with H_2_O_2_ (400 µM) for 6 h. The mRNA levels were evaluated by qPCR. **(E)** Western blot analysis. Densitometry analysis of each band (ratio of nuclear *p*-NFκB p-65/lamin B and cytoplasmic *p*-NFκB p-65/actin) was carried out with ImageJ program. **(F)** Representative blots of nuclear and cytoplasmic proteins. Each bar represents mean value ±SD (n = 5; each run in triplicate). Data were analyzed by one-way ANOVA and Tukey’s post hoc test for multiple comparisons. *p < 0.05 vs. control; †*p* < 0.05 vs. H_2_O_2_; ‡*p* < 0.05 vs. 1,25(OH)_2_D_3_ and MZ.

Furthermore, we analyzed IL-1β and TNF-α mRNA levels to assess the effect of 1,25(OH)_2_D_3_ and meso-zeaxanthin (MZ) in modulation of inflammatory response, in ARPE-19 cells challenged with H_2_O_2_ (400 µM) for 6 h. H_2_O_2_ challenge led to significant (p < 0.05) increase in IL-1β and TNF-α mRNA expression (Figures 4A,B). Treatment with 1,25(OH)_2_D_3_ (50 nM), MZ (0.1 µM), and their combination reverted the effect of H_2_O_2_ (Figures 4A,B). Furthermore, we assessed effects of those compounds in reducing MMP-9 and VEGF-A mRNA levels, both involved in retinal angiogenesis and neovascularization. H_2_O_2_ treatment induced a significant (*p* < 0.05) upregulation of both factors (Figures 4C,D). The MMP-9 mRNA levels were significantly (*p* < 0.05) reduced by 1,25(OH)_2_D_3_, MZ, and their combination, compared to H_2_O_2_-treated cells (Figure 4C). Only the combination of 1,25(OH)_2_D_3_ and MZ significantly reduced VEGF-A mRNA levels, in comparison to cells exposed to H_2_O_2_ (*p* < 0.05) (Figure 4D). Furthermore, we assessed the effect of tested compounds in terms of p65-NFκB nuclear translocation. H_2_O_2_ challenge led to a higher (*p* < 0.05) p-p65 nuclear translocation after 4 h. This process was significantly (*p* < 0.05) counteracted by pretreatment with 1,25(OH)_2_D_3_ (50 nM), MZ (0.1 µM), and their combination. Particularly, the combo significantly inhibited p65-NFκB translocation, compared to tested compounds and H_2_O_2_-exposed cells (Figures 4E,F).

### LPS Insult

**FIGURE 5:**
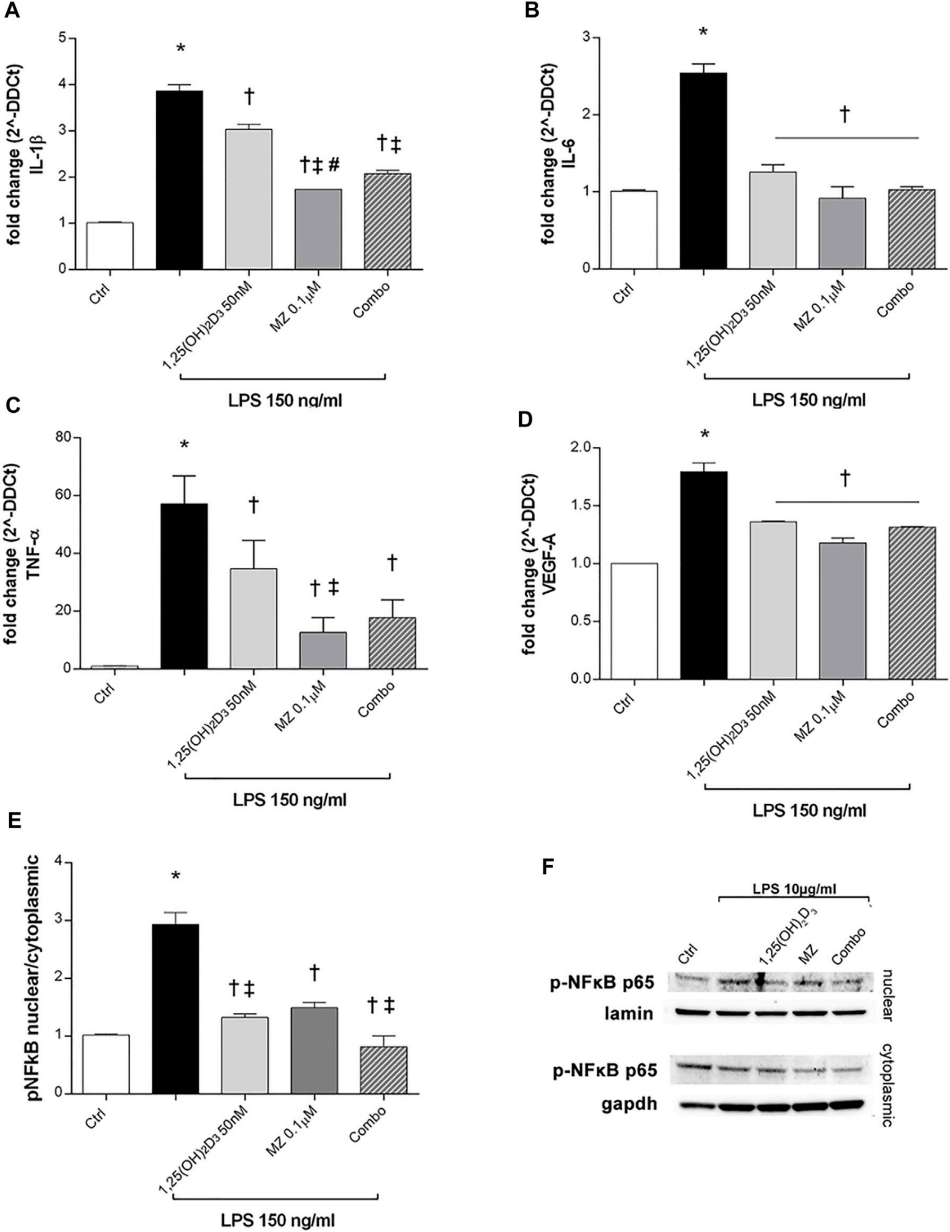
1,25(OH)_2_D_3_, meso-zeaxanthin (MZ), and their combination protect ARPE-19 cells from LPS-induced damage. 1,25(OH)_2_D_3_, meso-zeaxanthin (MZ), and their combo reduced IL-1β **(A)**, IL-6 **(B)**, TNF-α **(C)**, and VEGF-A **(D)** mRNA expression. ARPE-19 cells were pretreated for 24 h with 1,25(OH)_2_D_3_ (50 nM), MZ (0.1 µM), and their combo (1,25(OH)_2_D_3_ 50 nM + MZ 0.1 µM), and then challenged with LPS (150 ng/ml) for 2 h. The mRNA levels were evaluated by qPCR. **(E)** Densitometry of *p*-NFκB p65 nuclear translocation in treated cells. ARPE-19 cells were pretreated for 24 h with 1,25(OH)_2_D_3_ (50 nM), MZ (0.1 µM), and their combo (1,25(OH)_2_D_3_ 50 nM + MZ 0.1 µM), and then challenged with LPS (10 μg/ml) for 2 h. **(F)** Representative images of blots of nuclear and cytoplasmic protein. Each bar represents the mean value ±SD (*n* = 5; each run in triplicate). Data were analyzed by one-way ANOVA and Tukey’s post hoc test for multiple comparisons. *p < 0.05 vs. control; †*p* < 0.05 vs. LPS; ‡*p* < 0.05 vs. 1,25(OH)_2_D_3_ and MZ; #*p* < 0.05 vs. combo.

ARPE-19 cells were pretreated with 1,25(OH)_2_D_3_ (50 nM), meso-zeaxanthin (MZ, 0.1 µM), and their combination (combo: 1,25(OH)_2_D_3_ 50 nM + MZ 0.1 µM) for 24h, and then exposed to LPS (150 ng/ml) for 2 h. IL-1β, IL-6, and TNF-α mRNA levels were significantly increased in the LPS-stimulated cells, compared to control cells (*p* < 0.05). Both compounds and their combination (*p* < 0.05) significantly reduced cytokine mRNA levels (Figures 5A–C). MZ significantly reduced TNF-α mRNA expression, compared to 50 nM 1,25(OH)_2_D_3_ and the combo (50 nM 1,25(OH)_2_D_3_ + 0.1 µM MZ). Furthermore, LPS treatment significantly induced the upregulation of VEGF-A mRNA (*p* < 0.05) (Figure 5D), and the treatment with 1,25(OH)_2_D_3_, MZ, and their combo significantly reduced the expression of the latter (*p* < 0.05). After 2 h exposure, LPS (10 μg/ml) led to a significant increase of *p*-NFκB p65 nuclear translocation, in comparison to control cells (*p* < 0.05) (Figures 5E,F). The treatment with 1,25(OH)_2_D_3_, MZ, and their combo significantly inhibited this translocation, leading to a reduction in p-p65 nuclear protein amount (*p* < 0.05) (Figures 5E,F).

### TNF-α Insult

**FIGURE 6:**
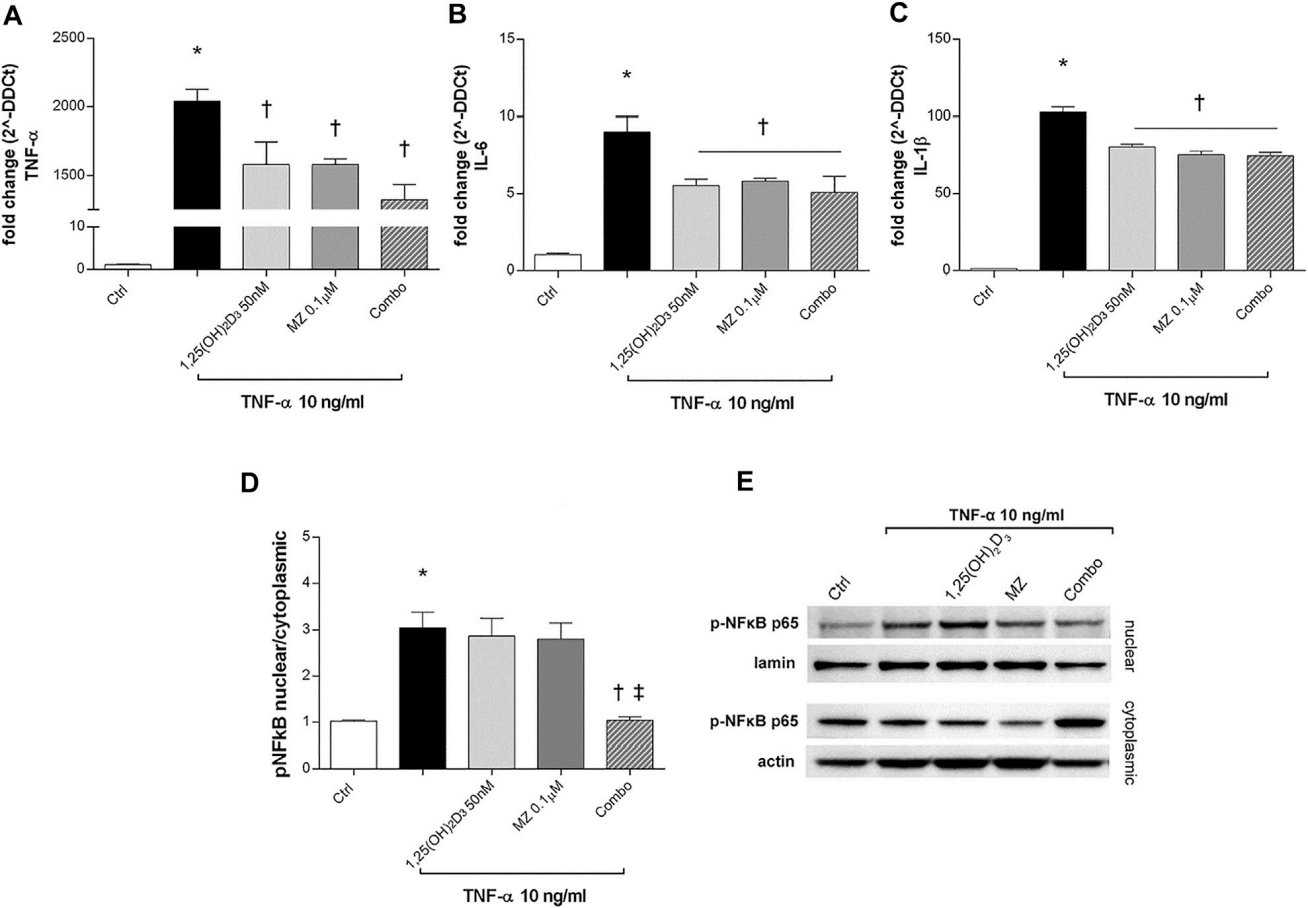
1,25(OH)_2_D_3_, meso-zeaxanthin (MZ), and the combination inhibit LPS-induced inflammation. 1,25(OH)_2_D_3_, meso-zeaxanthin (MZ), and their combo reduced TNF-α **(A)**, IL-6 **(B)**, and IL-1β **(C)** mRNA expression. ARPE-19 cells were pretreated for 24 h with 1,25(OH)_2_D_3_ (50 nM), MZ (0.1 µM), and their combo (1,25(OH)_2_D_3_ 50 nM + MZ 0.1 µM), and then challenged with TNF-α (10 ng/ml) for 2h, both for mRNA and protein analyses. The mRNA levels were evaluated by qPCR. **(D)** Densitometric analysis of each band (ratio of nuclear *p*-NFκB p65/lamin B and cytoplasmic *p*-NFκB p65/actin) was carried out with the ImageJ program. **(E)** Representative images of nuclear and cytoplasmic proteins. Each bar represents the mean value ±SD (*n* = 5; each run in triplicate). Data were analyzed by one-way ANOVA and Tukey’s post hoc test for multiple comparisons. *p < 0.05 vs. control; †*p* < 0.05 vs. TNF-α; ‡*p* < 0.05 vs. 1,25(OH)_2_D_3_ and MZ.

To evaluate ARPE-19 cells response to TNF-α challenge (10 μg/ml), we analyzed TNF-α, IL-6, and IL-1β mRNA levels. After 2 h, those cytokines were significantly increased by TNF-α treatment (10 ng/ml) (*p* < 0.05) and were strongly downregulated by 1,25(OH)_2_D_3_, meso-zeaxanthin (MZ), and the combo pretreatments (*p* < 0.05) (Figures 6A–C). We confirmed the anti-inflammatory effects of tested compounds against TNF-α exposure also through evaluation of the *p*-NFκB p65 nuclear translocation (Figures 6D,E). TNF-α challenge significantly increased the nuclear translocation of p-NFκB p65 (p < 0.05). Only the combination of 1,25(OH)_2_D_3_ and MZ significantly reduced the amount of nuclear *p*-NFκB p65 (*p* < 0.05) (Figures 6D,E).

### Bioinformatic Analysis

**FIGURE 7:**
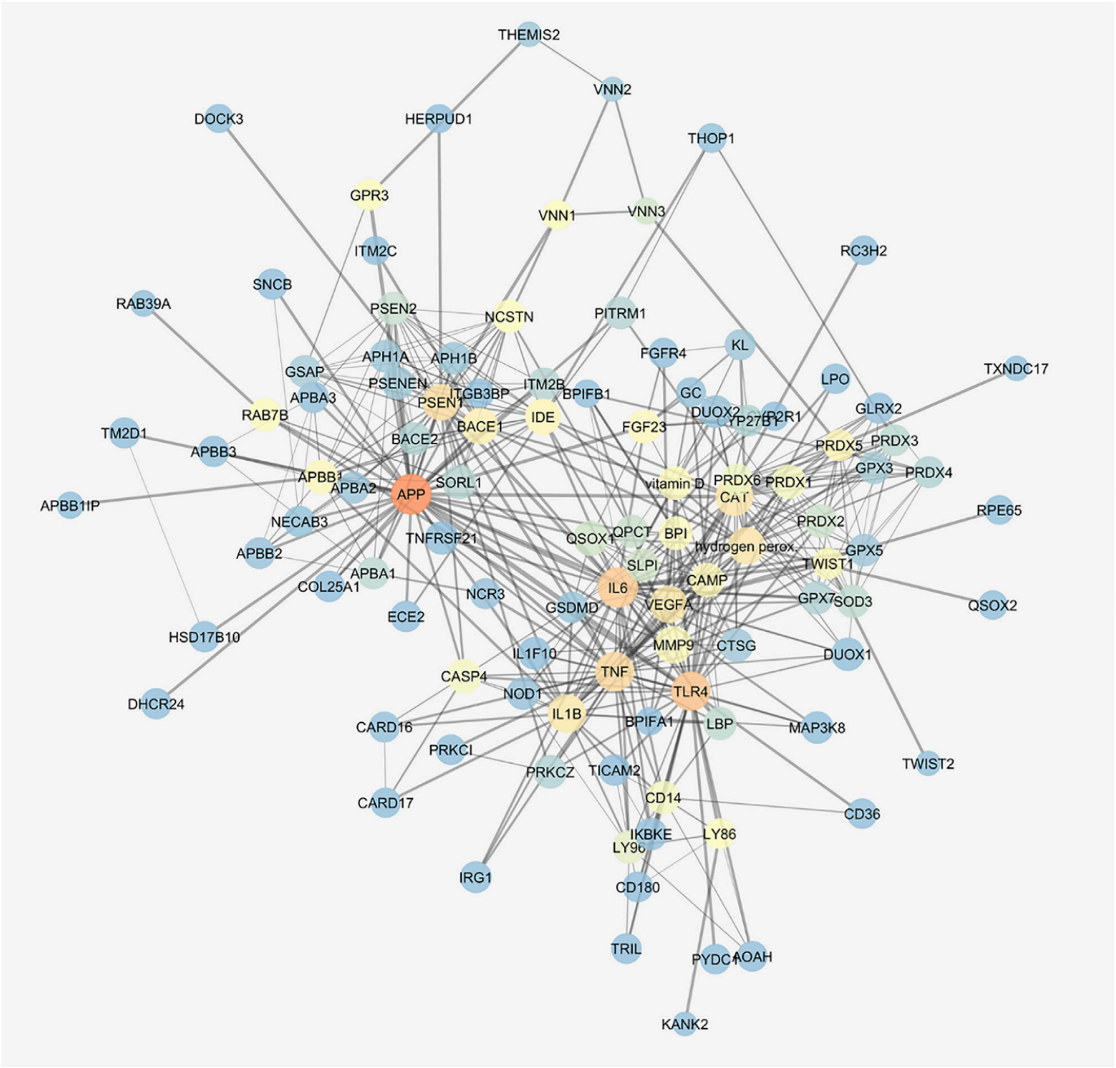
Gene activated by amyloid β, H_2_O_2_, and LPS are connected. STITCH protein–compound network representing the *in vitro* models. Nodes are represented on the basis of betweenness centrality values (color scale blue < red) and closeness centrality values (node dimension); edge thickness is proportional to edge betweenness values.

We built the protein–compound interaction network that resembled our integrated *in vitro* model of AMD through the STITCH compound app of Cytoscape v. 3.7.0, according to the approach described in the Methods section. The network was characterized by 136 nodes and 463 edges; a centrality metrics analysis was carried out treating the network as an indirect graph. Nodes with highest betweenness centrality have represented using a color scale (blue < red) (Figure 7), and the following nodes were characterized by the highest betweenness centrality and the average shortest path: APP > TLR4> IL6> TNF-α> PSEN1> H_2_O_2_> CAT > IL-1β, VEGF-A. We identified in this network functional clusters associated to the *in vitro* models used in our study: amyloid β (Supplementary Figure S1), H_2_O_2_ (Supplementary Figure S2), and inflammation, that is, LPS (Supplementary Figure S3) and TNF-α challenge (Supplementary Figure S4).

**FIGURE 8:**
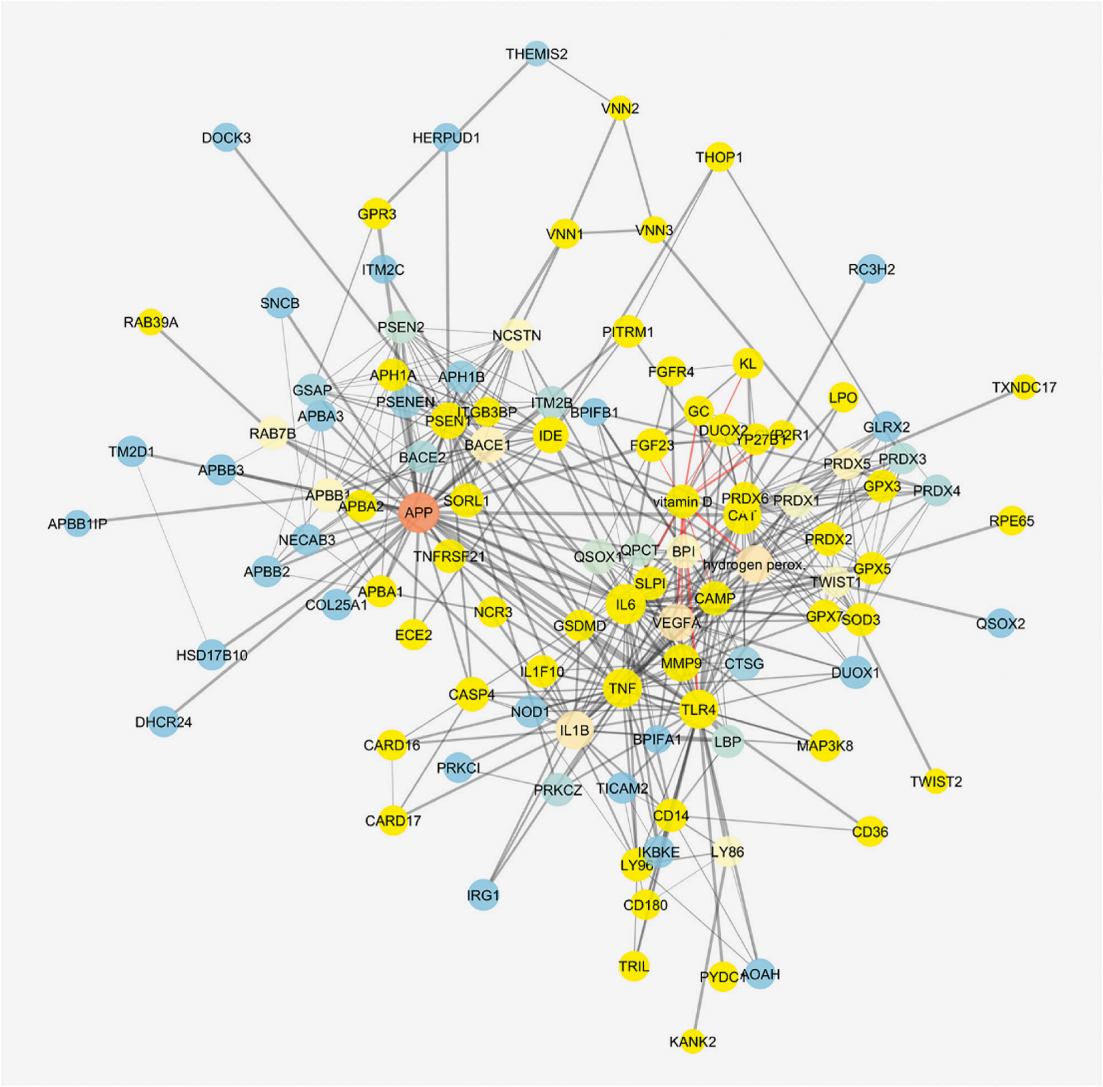
Vitamin D_3_–interacting nodes cover most of the STITCH protein–compound network. Red edges highlight direct interactions with vitamin D_3_, and yellow nodes represent direct and indirect interactors of vitamin D_3_.

The cluster related to vitamin D_3_ covered most of the network (Figure 8), but meso-zeaxanthin was linked only to RPE65 and VEGF-A. This last result would be linked to lack of literature data on meso-zeaxanthin, beyond compound antioxidant properties, and the documented RPE65 “lutein to meso-zeaxanthin” isomerase activity (Shyam et al., 2017).

## Discussion

Although several pathogenic mechanisms have been linked to onset and progression of AMD, management, and treatment of AMD is still affected by several unmet medical needs. Specifically, only wet AMD could be therapeutically managed through costly and invasive treatments, such as the anti-VEGF intravitreal injections, which can be ineffective in about 15% of patients (Krebs et al., 2013). Non-responders to intravitreal anti-VEGF treatments can encounter to irreversible vision loss, leading to burden of care linked to direct and indirect costs of blindness. Moreover, no therapy has been already approved for treatment of dry AMD, or for treatment of early phases of the disease.

Multivitamins and mineral supplementation are largely marketed for AMD patients, and clinical trials were carried out regarding specific formulations; the first was the Age-Related Eye Disease Study (2001) formulation, containing vitamins C and E, beta-carotene, and zinc with copper (Age-Related Eye Disease Study Research Group 2001; Kassoff et al., 2001; Chew et al., 2013a, 2014). A second trial “The Age-Related Eye Disease Study 2” (AREDS2) evidenced that substitution of β-carotene with lutein/zeaxanthin was safer for smokers and former smokers. In this AREDS2 study, lutein or zeaxanthin was compared with placebo. The authors found that there was a modest or no effect on AMD progression, but this was not statistically significant since all participants took the AREDS formula, and there was no proper control group (Chew et al., 2013b). On this regard, a systematic review with a meta-analysis evidenced that lutein and zeaxanthin supplements have little or no effect in AMD progression (Evans and Lawrenson, 2017), although this conclusion had a low level of certainty. In the same systematic review, authors evidenced that AMD subjects taking antioxidants multivitamin supplementation, including vitamin D_3_, were at lower risk of AMD progression, but no evidence on visual acuity was found by meta-analysis. Since, there is no intervention to slow down the progression of the disease, depending on the AMD stage, correct supplementation of antioxidants and vitamins would be of benefit, but up to now, current supplement formulation trials did not provide evidence-based efficacy.

Therefore, in search of an improved formulation of supplements, we hereby explored for the first time, in an integrated *in vitro* model of AMD, the effects of 1,25(OH)_2_D_3_ (vitamin D_3_) and meso-zeaxanthin combination on several endpoints related to inflammation, oxidative stress, and cellular damage: amyloid β, H_2_O_2_, and inflammatory insults, that is, LPS and TNFα. The rationale of these integrated *in vitro* models of AMD is behind its multifactorial pathogenic etiology (Bucolo et al., 2006; Di Filippo et al., 2014; Fisichella et al., 2016; Platania et al., 2017, 2019; Romano et al., 2017; Giordano et al., 2020; Micera et al., 2021), involving amyloid-β and oxidative stress, has already been mentioned. As regards as, the LPS challenge is widely used as an experimental model of AMD, involving the activation of Toll-like receptor 4 (TLR-4) and the downstream activation of NFκB (Sung et al., 2019; Hikage et al., 2021), and then triggering the expression of inflammatory cytokines. While, the most potent downstream inflammatory cytokine, TNF-α has been found to promote, in ARPE-19 cells, secretion of proteins involved in AMD pathology, such as complement C3 (An et al., 2008). Worthy of note, antioxidant and anti-inflammatory strategies have been largely explored for treatment of ocular diseases (Bucolo et al., 1999; Shafiee et al., 2011).

As shown by our data, vitamin D_3_ and meso-zeaxanthin combination effectively protected cells from damage induced by β-amyloid, H_2_O_2_, LPS, and TNF-α. However, based on analyzed endpoints, we cannot hypothesize an additive or synergistic effect between vitamin D_3_ and meso-zeaxanthin. Specifically, the combination of vitamin D_3_ and meso-zeaxanthin was significantly effective, compared to the two single components, in decreasing Il-1β, TNF-α, and VEGF-A (H_2_O_2_ insult). Moreover, the combination of 1,25(OH)_2_D_3_ + meso-zeaxanthin, compared to the two single components, significantly reduced NFκB nuclear translocation, in ARPE-19 cells challenged with H_2_O_2_, LPS, and TNF-α. While in the β-amyloid model, both vitamin D_3_ and the combination with meso-zeaxanthin inhibited NFκB pathway activation but not the meso-zeaxanthin treatment alone.

Our findings about vitamin D_3_ activity on ARPE-19 cells challenged with H_2_O_2_ and LPS are supported by recent findings on 1,25(OH)_2_D_3_ antioxidant and anti-inflammatory activity (Fernandez-Robredo et al., 2020; Hernandez et al., 2021). Preclinical and clinical studies evidenced protective effects of vitamin D_3_ in Alzheimer disease, an amyloid-β–related pathology (Sultan et al., 2020; McCarty et al., 2021). The link between AMD and AD pathology has been largely documented (Romano et al., 2017), and low-vitamin D_3_ levels in serum were linked to progression of AMD, however with small effect (i.e., small adjusted odd ratio) (McKay et al., 2017). We proved for the first time that in ARPE-19 cells, vitamin D_3_, meso-zeaxanthin, and their combination protected cells from damage induced by β-amyloid exposure, oxidative stress, and inflammatory stimuli. Recently, it has been demostrated that lutein and meso-zeaxanthin are taken up by ARPE-19 cells via different mechanisms with preferential uptake of meso-zeaxanthin (Thomas and Harrison, 2016). Additionally, it is known that the enzyme RPE65 converts dietary lutein to meso-zeaxanthin in the retinal pigment epithelium of vertebrates (Shyam et al., 2017). Meso-zeaxanthin is a well-known antioxidant compound that accumulates as other xanthophyll carotenoids in the macula, increasing macular pigments and then protecting pigmented epithelial cells and photoreceptors from photo-oxidative stress (Bone et al., 2007). Up to now, there is an evidence of non-inferiority of meso-zeaxanthin enriched formulation, compared to AREDS2 formulation (Akuffo et al., 2017). On the contrary, non-advanced AMD subjects taking the meso-zeaxanthin–enriched formulation have shown significant higher meso-zeaxanthin and zeaxanthin serum levels and total serum carotenoids, than AREDS2 subjects (Akuffo et al., 2017). Despite large-scale clinical trials that showed the benefits of xanthophyll carotenoids against AMD, recommendations for nutritional interventions are underappreciated by ophthalmologists. Besides the well-known antioxidant activity of meso-zeaxanthin, only few non-ocular studies have reported an anti-inflammatory activity (Firdous et al., 2015; Sahin et al., 2017). Lack of literature findings about meso-zeaxanthin’s anti-inflammatory activity was also emerged in our *in silico* analysis. Interestingly, meso-zeaxanthin decreased levels of nuclear p-NFκB and TNF-α secretion in the insulin-resistant rodent model (Sahin et al., 2017); this anti-inflammatory activity has been evidenced also in our experimental settings, since the single treatment with meso-zeaxanthin effectively delivered anti-inflammatory effects.

In conclusion, we hereby provided *in vitro* evidence that vitamin D_3_ and meso-zeaxanthin association protected retinal pigmented epithelium from several damages that recapitulate the multifactorial pathogenic mechanisms of AMD. With this regard, vitamin D_3_ and meso-zeaxanthin supplementation would be of value in AMD patients, especially for subject diagnosed with early diagnosis of AMD, as already evidenced by several systematic reviews.

## Data Availability

The original contributions presented in the study are included in the article/Supplementary Material; further inquiries can be directed to the corresponding author.
