# Supplementary material for: Effects of Vitamin D3 and Meso-Zeaxanthin on Human Retinal Pigmented Epithelial Cells in Three Integrated in vitro Paradigms of Age-Related Macular Degeneration
Source: Front Pharmacol. 2021 Nov 5;12:778165. doi: 10.3389/fphar.2021.778165 (PMC8602342; doi:10.3389/fphar.2021.778165)
Supplement: Supplementary file 1 [file DataSheet1.docx]

**SUPPLEMENTARY MATERIAL**

**Effects of vitamin D_3_ and meso-zeaxanthin combination on human retinal pigmented epithelial cells in three integrated in-vitro paradigms of age-related macular degeneration**

**Francesca Lazzara^1^, Federica Conti^1^, Chiara Bianca Maria Platania^1^, Chiara M. Eandi^2,3^, Filippo Drago^1^ and Claudio Bucolo^1*^**

^1^ Department of Biomedical and Biotechnological Sciences, School of Medicine, University of Catania, Catania, Italy; ^2^Department of Ophthalmology, University of Lausanne, Fondation Asile des Aveugles, Jules Gonin Eye Hospital, Lausanne, Switzerland; ^3^Department of Surgical Sciences, University of Torino, Italy


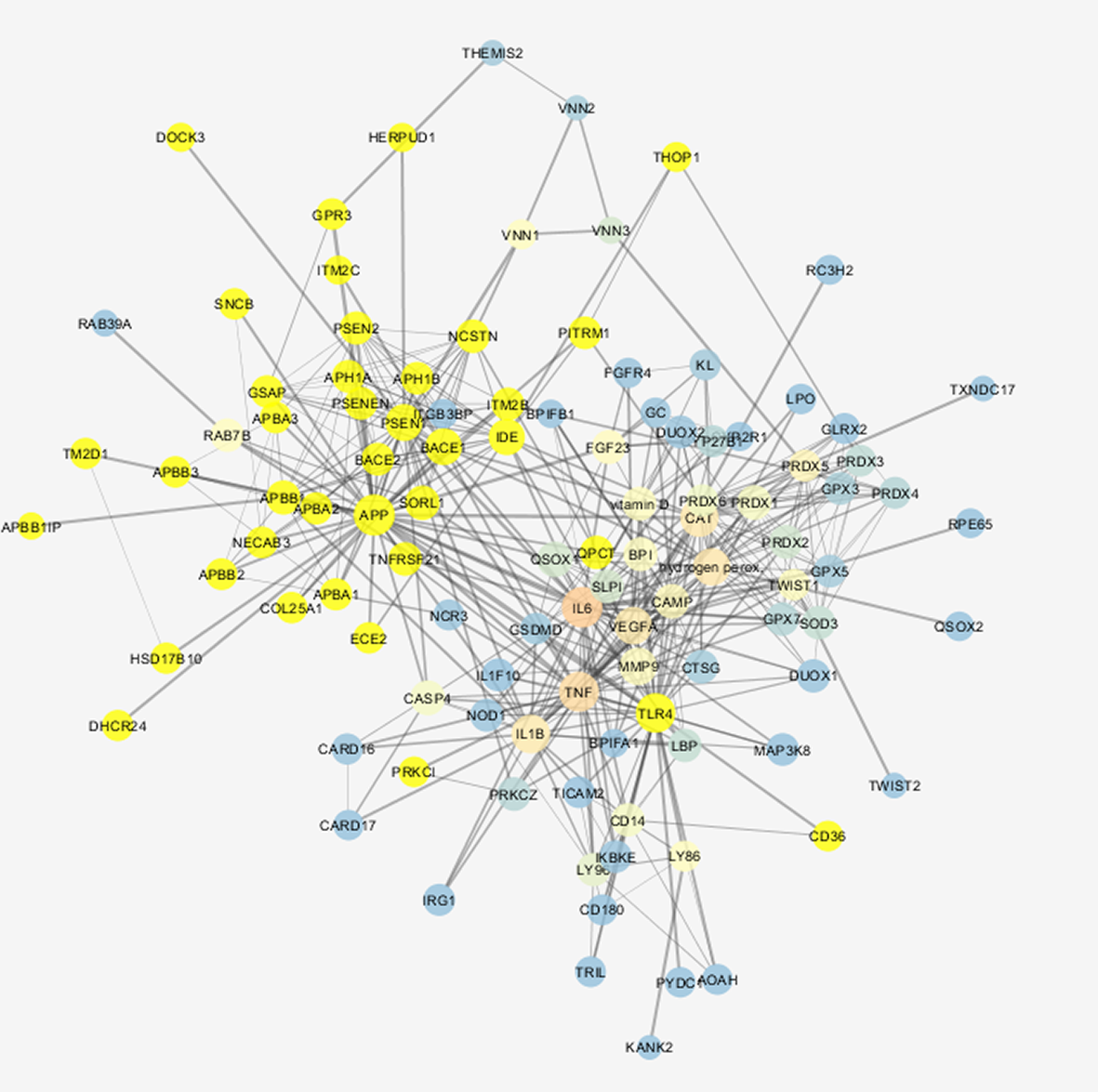


**Figure 1S.** Amyloid β cluster in the STITCH protein-compound network. Yellow nodes represent direct and indirect interactors of amyloid β node.

**
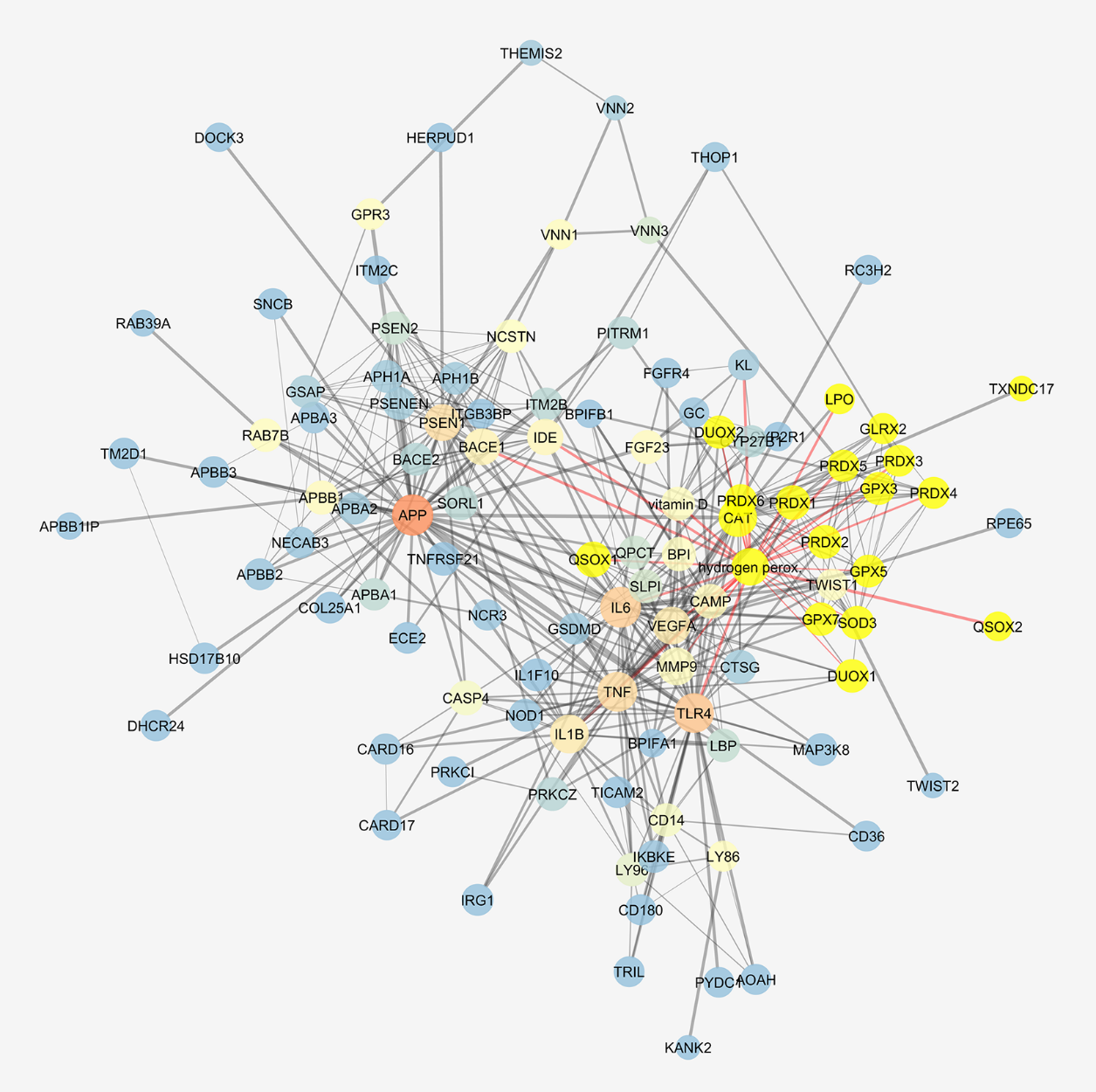
**

**Figure 2S:** Hydrogen peroxide (H_2_O_2_) interacting nodes in the STITCH protein-compound network. Red edges highlight direct interactions with H_2_O_2_, yellow nodes represent direct and indirect interactors of H_2_O_2_.


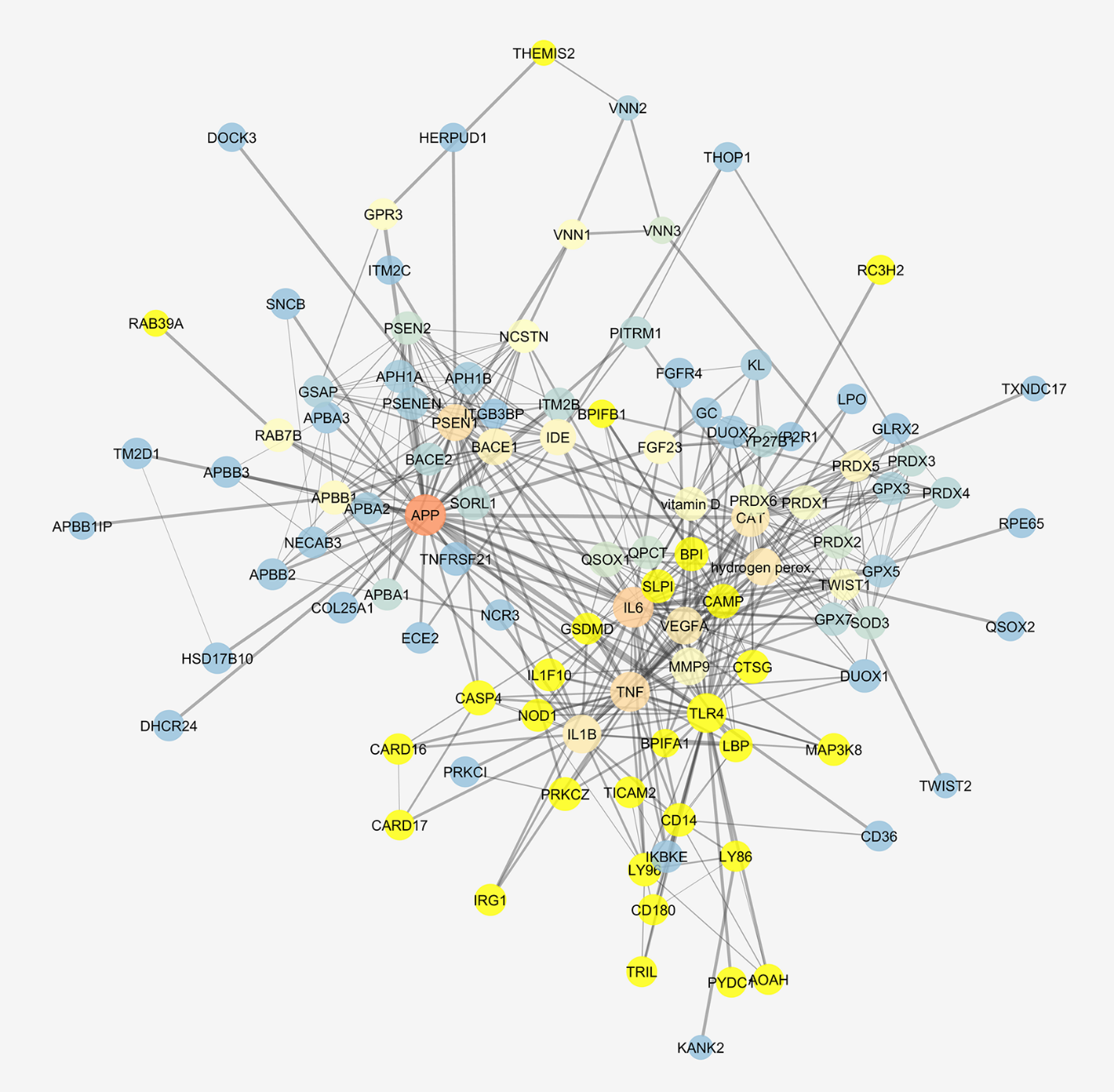


**Figure 3S:** LPS interacting nodes in the STITCH protein-compound network. Yellow nodes represent direct and indirect interactors of LPS.


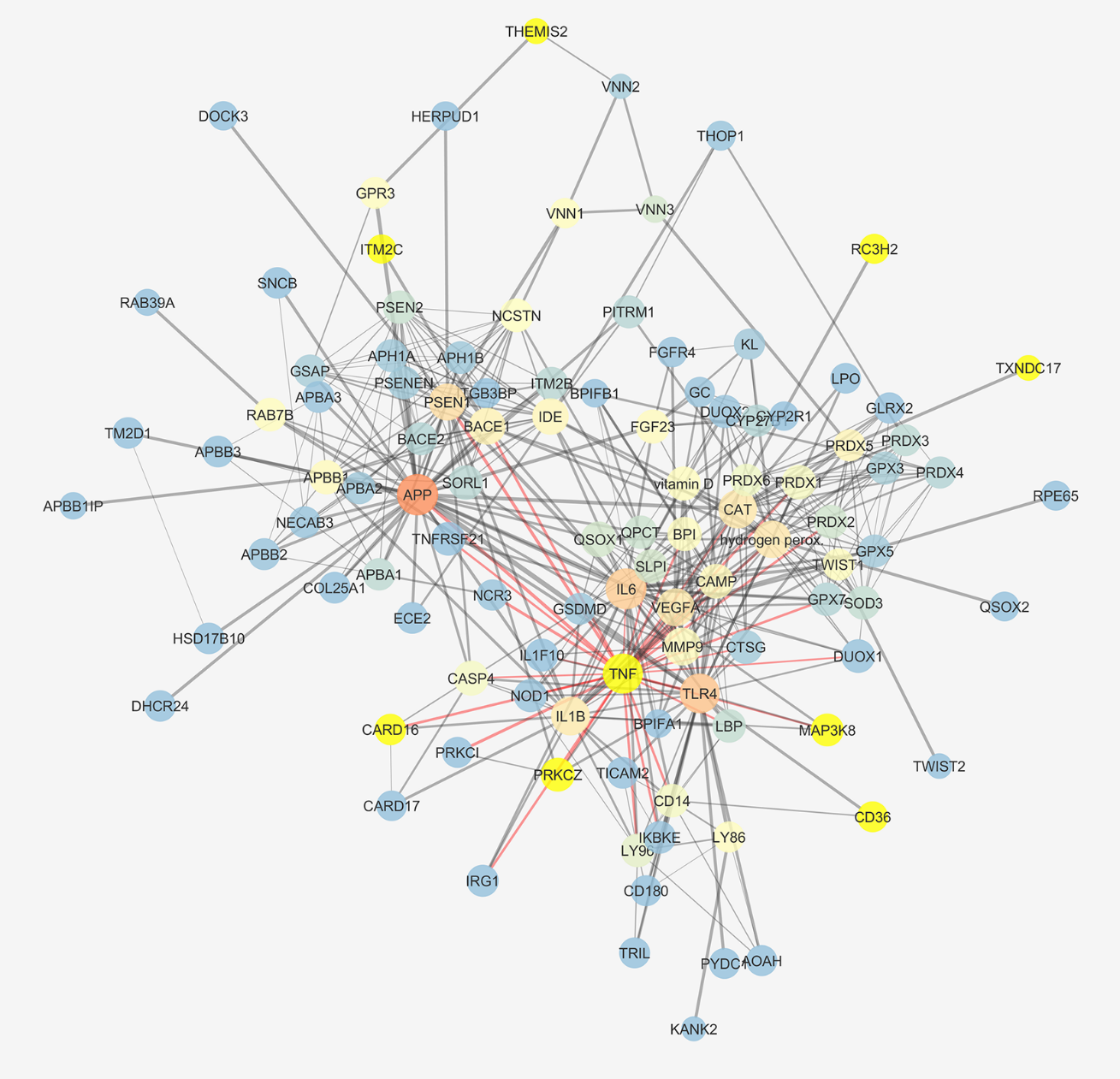


**Figure 4S:** TNFα interacting nodes in the STITCH protein-compound network. Red edges highlight direct interactions with TNFα, yellow nodes represent direct and indirect interactors of TNFα.
